# Tetramethylpyrazine and Paeoniflorin Inhibit Oxidized LDL-Induced Angiogenesis in Human Umbilical Vein Endothelial Cells via VEGF and Notch Pathways

**DOI:** 10.1155/2018/3082507

**Published:** 2018-11-21

**Authors:** Rong Yuan, Weili Shi, Qiqi Xin, Binrui Yang, Maggie Puiman Hoi, Simon Mingyuan Lee, Weihong Cong, Keji Chen

**Affiliations:** ^1^Laboratory of Cardiovascular Diseases, Xiyuan Hospital, China Academy of Chinese Medical Sciences, Beijing 100091, China; ^2^Graduate School, Beijing University of Chinese Medicine, Beijing 100029, China; ^3^State Key Laboratory of Quality Research in Chinese Medicine, Institute of Chinese Medical Sciences, University of Macau, Macau

## Abstract

Atherosclerotic plaque angiogenesis is key factor in plaque instability and vulnerability, and low concentrations of oxidized low density lipoprotein (ox-LDL) promote the in vitro angiogenesis of endothelial cells and play an important role in plaque angiogenesis. Ligusticum chuanxiong Hort. and Radix Paeoniae Rubra herb pair in Chinese medicine obtains the optimum therapeutic efficacy in atherosclerosis, and their major active ingredients tetramethylpyrazine (TMP) and paeoniflorin (PF) are reported to alleviate atherosclerosis. The aim of this study was to investigate the effects of TMP and PF on ox-LDL-induced angiogenesis and the underlying mechanism. Human umbilical vein endothelial cells (HUVECs) were incubated with ox-LDL and were then treated with TMP, PF, or a combination of TMP and PF. Cell proliferation, migration, tube formation, and the expression of angiogenesis-related proteins were measured. Synergism was evaluated using the combination index in cell proliferation. We found that TMP and PF attenuated the in vitro angiogenesis in ox-LDL-induced HUVECs. In addition, the combination of TMP and PF not only inhibited the ox-LDL-induced expression of CD31, vascular endothelial growth factor (VEGF), and VEGF receptor 2 (VEGFR2) but also decreased the ox-LDL-induced expression of Notch1, Jagged1, and Hes1. In summary, the combination of TMP and PF suppresses ox-LDL-induced angiogenesis in HUVECs by inhibiting both the VEGF/VEGFR2 and the Jagged1/Notch1 signaling pathways, which might contribute to the stability of plaques in atherosclerosis.

## 1. Introduction

Acute coronary syndrome is often related to atherosclerotic plaque rupture and thrombosis, while angiogenesis is a key factor in plaque destabilization leading to rupture [1, 2]. Angiogenesis is a complex process that involves cell proliferation, migration, basement membrane degradation, and neovessel organization and maturation. Several studies suggest that angiogenesis contributes to the growth of atherosclerotic lesions and plaque destabilization by aggravating inflammation-related injury and causing intraplaque hemorrhage [3–5]. Oxidized low density lipoprotein (ox-LDL) is, at least in part, responsible for angiogenesis in atherosclerotic regions [6]. Previous studies have demonstrated that low concentrations of ox-LDL promote angiogenesis in human endothelial cells, thus leading to plaque vulnerability and intravascular thrombosis [7–10]. Therefore, the inhibition of angiogenesis has been considered as a potential therapeutic target in atherosclerosis [11].

Many leading researchers have advocated using combination approaches to pursue the optimum therapeutic efficacy and to improve the patient's health status [12]. Ligusticum chuanxiong Hort. and Radix Paeoniae Rubra have been used for many years in traditional Chinese medicine as an herb pair to treat atherosclerotic diseases and inflammatory problems, and the combination of these two drugs achieves optimum therapeutic efficacy. It is reported that the compound of active constituents of Ligusticum chuanxiong Hort. and Radix Paeoniae Rubra can stabilize plaques and inhibit angiogenesis in plaque lesions [13, 14]. Tetramethylpyrazine (TMP) is the active ingredient of Ligusticum chuanxiong Hort., which could attenuate atherosclerosis development and protect endothelial cells [15]. Paeoniflorin (PF) is the active ingredient of Radix Paeoniae Rubra, which could inhibit cell proliferation and alleviate atherosclerosis [16, 17]. However, the effect of TMP and PF on ox-LDL-induced angiogenesis has not been studied.

Combination therapy and synergistic analysis have been used to investigate herb pairs in Chinese medicine [18]. Therefore, we sought new strategies of combining TMP with PF to perform the synergistic analysis and then to observe whether they exhibit inhibitory effect on ox-LDL-induced angiogenic properties in human umbilical vein vascular endothelial cells (HUVECs). Moreover, the underlying mechanism of angiogenesis with combined treatment was also investigated.

Vascular endothelial growth factor (VEGF) is a key proangiogenic factor that promotes intraplaque angiogenesis. VEGF signaling is predominately mediated through the activation of VEGF receptor 2 (VEGFR2) on endothelial cells, which stimulates cell proliferation and migration and thus promotes the formation of new vessels; in turn, these events can induce plaque progression and eventual hemorrhage [19, 20]. In addition, the Notch signaling pathway plays a significant role in angiogenesis. During Notch activation, the upregulation of Jagged1 signaling in endothelial cells promotes the nuclear translocation of the Notch1 intracellular domain, which is the biologically active signal transducer [21]. Jagged-dependent Notch signal activation promotes pathological angiogenesis [22]. However, little is known about whether these two pathways can be regulated by the individual or combined administration of TMP and PF.

In this study, we intend to determine the effect of TMP and PF on ox-LDL-induced angiogenesis and whether the VEGF and Notch pathways were regulated by TMP and PF in ox-LDL-induced HUVECs.

## 2. Materials and Methods

### 2.1. Materials and Reagents

TMP hydrochloride and PF were purchased from Shanghai Yuanye Bio-Technology Co., Ltd. (Shanghai, China). Ox-LDL was purchased from Beijing Solarbio Science & Technology Co., Ltd. (Beijing, China). Endothelial Cell Medium (ECM) was purchased from Scien Cell Research Laboratories (CA, USA). ELISA kits for VEGF were purchased from Multi Sciences (Hangzhou, China). Primary antibodies against VEGFR2, Notch1, Jagged1, and Hes1 were purchased from Cell Signaling Technology (CA, USA). Primary antibodies against CD31 and vWF were purchased from Proteintech (Chicago, USA). Matrigel basement membrane matrix was purchased from Becton, Dickinson and Company (New Jersey, USA). L685458 was purchased from Med Chem Express (New Jersey, USA). Other reagents were of commercially available analytical grade.

### 2.2. Cell Culture and Induction

HUVECs were generated by extraction from human umbilical veins [23]. The cell line was cultured in ECM supplemented with 5% fetal bovine serum, 1% penicillin/streptomycin solution and 1% endothelial cell growth supplement at 37°C in an atmosphere with 5% CO_2_. HUVECs were then incubated with different concentrations (0, 1, 5, 10, 20, 40, or 80 *μ*g/mL) of ox-LDL for 12 h and 24 h. Then a proper concentration was used in subsequent experiments. TMP, PF, and L685458 (Notch inhibitor) were dissolved in DMSO to prepare stock solutions. Stock solutions were diluted further into cell culture medium immediately before use. The final concentration of DMSO was less than 0.2%.

### 2.3. Cell Proliferation

The ox-LDL-induced proliferation of HUVECs was measured using MTT assay [24]. Briefly, HUVECs were seeded in 96-well plates. After 24 h, cells were starved in low-serum medium (0.2%) for 12 h. Then, cells in the control group were treated with the vehicle (saline solution), whereas cells in the other groups were treated with ox-LDL for 24 h. Next, the control group and the ox-LDL-induced groups were treated with the vehicle, while the TMP and PF groups were treated with various concentrations of TMP (10, 1, 0.1, or 0.01 *μ*mol/L) or PF (10, 1, 0.1, or 0.01 *μ*mol/L), respectively. The combination groups were treated with TMP and PF at different concentration combinations. After drug stimulation for 24 h, the optical density (OD) was measured using a microplate reader (Epoch 2, BioTek Instruments, USA) after incubation with MTT solution for 4 h at 37°C followed by incubation with DMSO for 5 min. Each condition included replicate wells with at least three independent repeats.

### 2.4. Drug Combination Analysis

The effects of the combination of TMP and PF were analyzed by CompuSyn software as previously described [25, 26]. Effective rate data were acquired from the MTT assay, and the combination index (CI) values were generated for a range of fraction affected (Fa) levels from 0.05-0.9. The Fa-CI plot illustrates the numerical CI values at different levels. The CI value is a mathematical and quantitative representation of the pharmacological interplay of two drugs (CI>1, antagonism; CI=1, additive; CI<1, synergism).

### 2.5. Cell Migration

Cell migration was detected using a wound healing assay as described previously [27]. HUVECs were seeded in 48-well plates and incubated at 37°C for 24 h. Subsequently, HUVECs were induced with ox-LDL and treated with TMP and PF, either alone or in combination, for 12 h. The tested concentrations of TMP and PF were selected based on synergistic analysis in cell proliferation. Confluent HUVECs were scratched with a pipette tip, and images were obtained before drug intervention and 12 h after drug intervention. Migration was quantified as the difference between the width of the wound area covered with cells and the width of the cell-free wound area. All assays were repeated three times independently.

### 2.6. Cell Tube Formation

Endothelial-like tube formation was examined as described previously [28]. A Matrigel basement membrane matrix was added to 96-well plates and incubated at 37°C for 30 min to allow gel formation. Confluent HUVECs were harvested and diluted in 100 *μ*L of low-serum medium containing ox-LDL and drugs; cells were then seeded in the Matrigel basement membrane matrix-coated 96-well plates and incubated at 37°C for 8 h. Cells in the control group were treated with vehicle, whereas cells in the drug groups were treated with TMP and PF either alone or in combination. The network-like structures were examined under an inverted microscope. The number of branching points in 3 random fields per well was quantified by ImageJ software.

### 2.7. Immunofluorescence

The angiogenesis markers CD31 and vWF were examined as described previously [29]. HUVECs were seeded on coverslips in 12-well plates for 24 h and were then induced with ox-LDL for 24 h and subjected to drug intervention for 24 h. After drug stimulation, cells were fixed for 30 min with 4% formaldehyde, permeabilized with Triton X-100 for 5 min, and blocked for 60 min. CD31 antibodies (1:200) and vWF antibodies (1:100) were added for overnight incubation (4°C). Subsequently, cells were washed with PBS and incubated with the appropriate secondary antibody (FITC-conjugated goat anti-rabbit IgG and Cy3-conjugated goat anti-mouse IgG) for 1 h. Nuclei were stained with DAPI for 5 min. Images were obtained at random using a fluorescence microscope, and the integral OD (IOD) was calculated. Each experiment was repeated on at least three occasions.

### 2.8. ELISA

HUVECs were seeded in 25 cm^2^ culture flasks, induced with ox-LDL for 24 h, and subjected to treatment with TMP and PF, either alone or in combination, for 24 h. The cell supernatant was harvested, and VEGF concentrations were detected with ELISA kits according to the manufacturer's instructions.

### 2.9. Western Blotting

Proteins were detected as previously described [30, 31]. HUVECs were seeded in 25 cm^2^ culture flasks, induced with ox-LDL for 24 h, and treated with TMP and PF, either alone or in combination, for 24 h. Proteins were extracted and protein concentrations were quantified with a BCA Protein Assay kit according to the instructions. Protein (20 *μ*g) was separated by 8%-10% SDS-PAGE and then transferred to PVDF membranes. The membranes were blocked with 5% nonfat milk in TBST for 1 h at room temperature and were then probed with primary antibodies (dilution with 1:1000) overnight at 4°C. Following incubation with the corresponding secondary antibody and three washes in TBST, the protein blots were visualized using a Chemi Doc XRS system with Image Lab software (Bio-Rad Laboratories, CA, USA).

### 2.10. Administration of the Notch Inhibitor L685458

L685458 is a potent inhibitor of amyloid *β*-protein precursor *γ*-secretase activity in the Notch signaling pathway. Cells were pretreated with L685458 (10 *μ*mol/L) for 30 min and then stimulated with ox-LDL for 24 h. Subsequently, western blotting was employed to evaluate the treatment effect, and the details of all treatments are indicated in the figure legends.

### 2.11. Statistical Analysis

One-way analysis of variance (ANOVA) was used to evaluate the statistical significance of data among the groups. A* P*⩽0.05 was considered statistically significant. All of the data were analyzed with SPSS 17.0 software and are presented as the means ± SD.

## 3. Results

### 3.1. TMP and PF Inhibit Ox-LDL-Induced HUVEC Proliferation with Synergistic Effect

**Figure 1:**
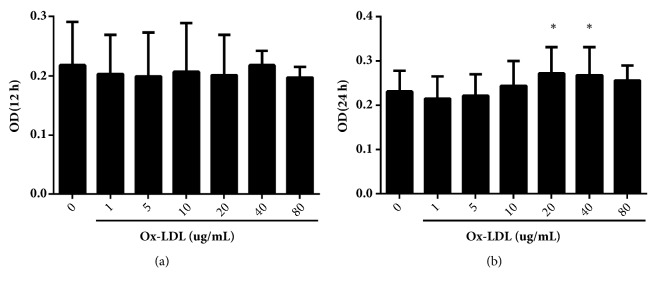
Ox-LDL-induced HUVEC proliferation at (a) 12 h and (b) 24 h. Values are means ± SD. *∗P* < 0.05 versus control group.

**Figure 2:**
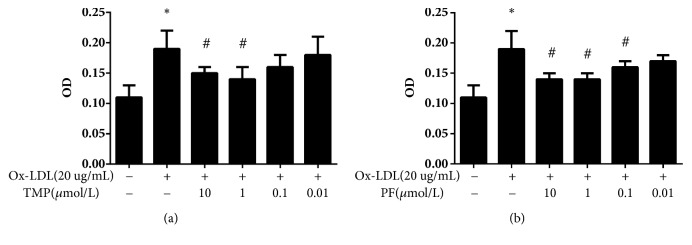
Effect of (a) TMP or (b) PF on ox-LDL-induced HUVEC proliferation. Values are means ± SD. *∗P* < 0.05 versus control group; #*P* < 0.05 versus ox-LDL-induced group.

**Figure 3:**
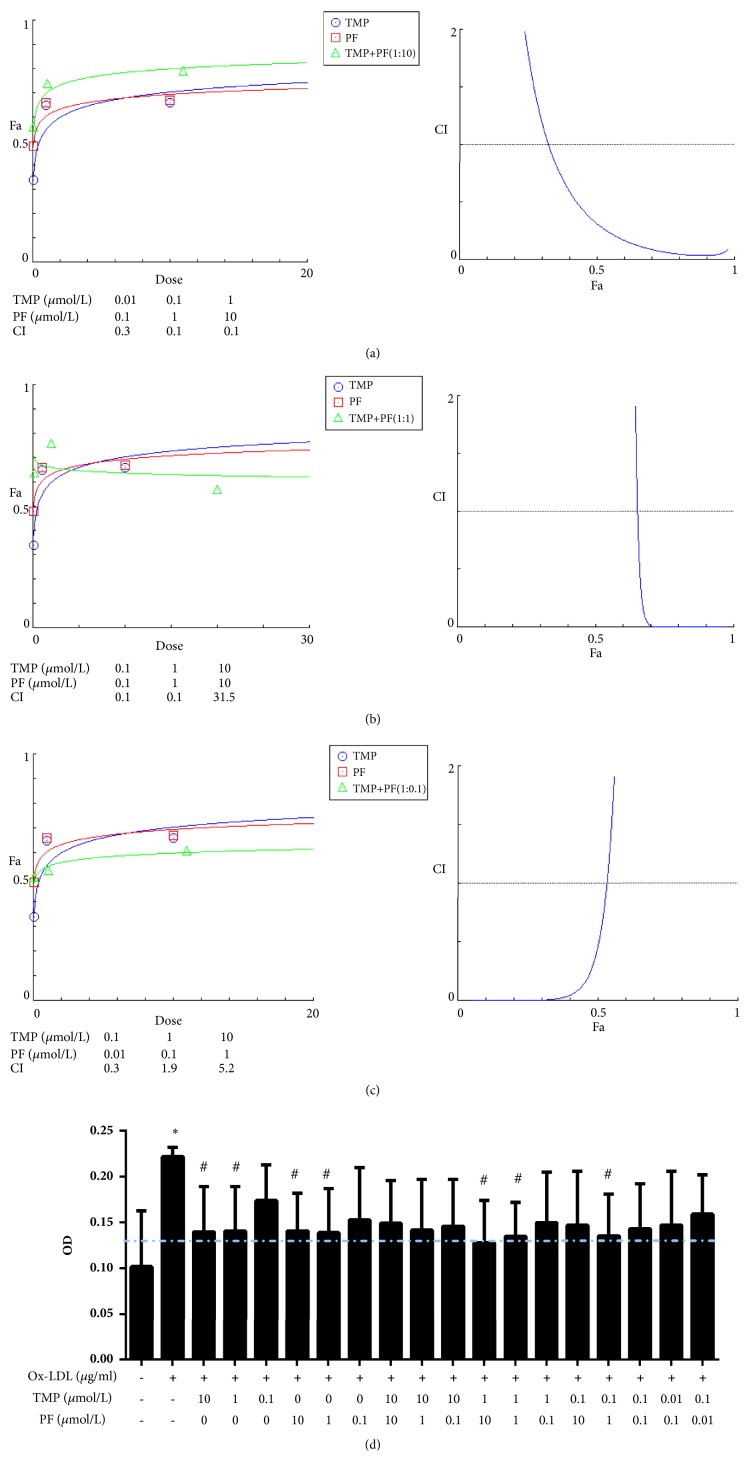
Combined effects of TMP and PF on ox-LDL-induced HUVEC proliferation. The CI values and Fa-CI plots generated using CompuSyn software for different concentration ratios of TMP and PF: (a) 1:10, (b) 1:1, and (c) 1:0.1. (d) Combined effects after treatment with 0, 0.01, 0.1, 1, or 10 *μ*mol/L TMP in combination with either 0, 0.01, 0.1, 1, or 10 *μ*mol/L PF for 24 h. Values are means ± S.D. *∗P* < 0.05 versus control group; #*P* < 0.05 versus ox-LDL-induced group.

In our results, 20 ug/ml ox-LDL enhanced HUVECs proliferation at 24 h when compared with control group (*P*<0.05, Figure 1), and this concentration was used for the subsequent experiments. After 24-h treatment, TMP alone (10 *μ*mol/L and 1 *μ*mol/L) or PF alone (10 *μ*mol/L, 1 *μ*mol/L, and 0.1 *μ*mol/L) inhibited cell proliferation when compared with ox-LDL-induced group (*P*<0.05, Figure 2). Then, HUVECs were treated with different effective concentrations of TMP and PF, either alone or in combination. All combinations of TMP and PF, except 10 *μ*mol/L TMP + 10 *μ*mol/L PF, 1 *μ*mol/L TMP + 0.1 *μ*mol/L PF, and 10 *μ*mol/L TMP + 1 *μ*mol/L PF, exerted synergistic effects (CI < 1) and had a CI value of less than 1 at most Fa levels (Figures 3(a)–3(c)). Specifically, TMP and PF (1:10) displayed more effective synergistic activity at different dose and better dose-response relationship than did TMP and PF administered in the other ratios. Furthermore, the combination of 1 *μ*mol/L TMP and 10 *μ*mol/L PF, 1 *μ*mol/L TMP and 1 *μ*mol/L PF, 0.1 *μ*mol/L TMP and 1 *μ*mol/L PF significantly inhibited cell proliferation (*P*<0.05), and the combination of 1 *μ*mol/L TMP and 10 *μ*mol/L PF showed the greatest inhibitory effect (Figure 3(d)); thus, this concentration was used for the subsequent experiments.

### 3.2. TMP and PF Inhibit Ox-LDL-Induced HUVEC Migration

**Figure 4:**
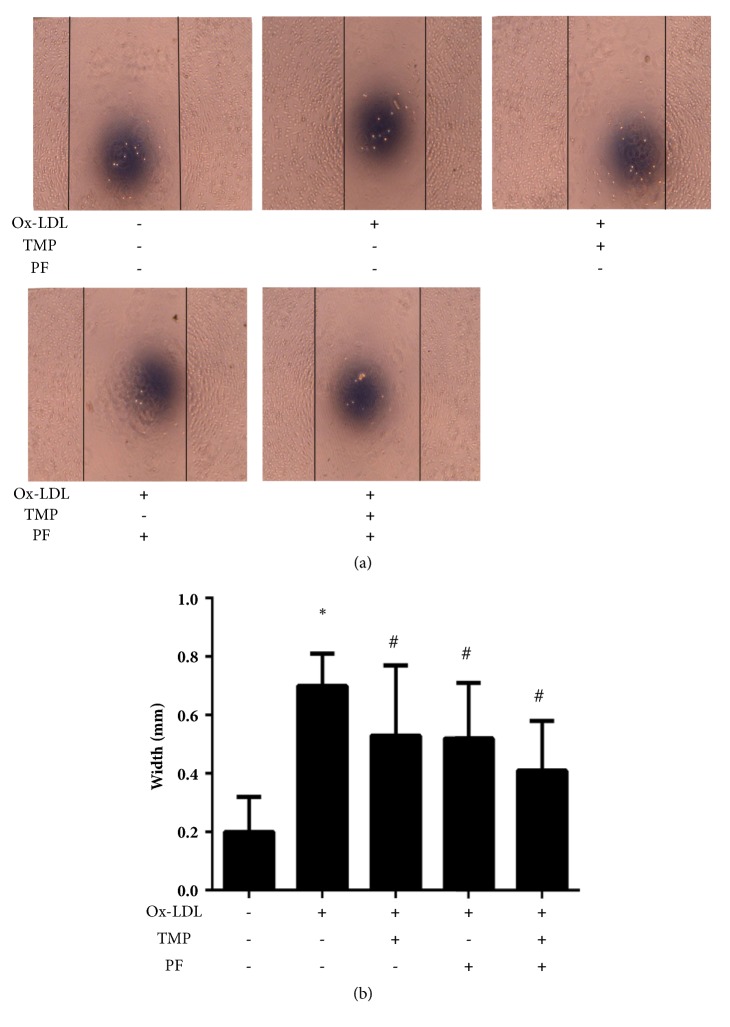
Effects of TMP and PF alone or in combination on ox-LDL-induced HUVEC migration. (a) The scratches were examined under an inverted microscope after 12 h (10×). (b) Quantitative analysis of the migration width. Values are means ± SD. *∗P *< 0.05 versus control group; #*P* < 0.05 versus ox-LDL-induced group.

Ox-LDL intensively promoted the HUVEC migration when compared with control group (*P*<0.05), while TMP and PF, either alone or in combination, suppressed the HUVEC migration when compared with ox-LDL-induced group (*P*<0.05). Moreover, combination treatment with TMP and PF showed the strongest inhibitory effect (Figure 4). These results indicated that TMP and PF, either alone or in combination, exhibited inhibitory effects on ox-LDL-induced HUVEC migration.

### 3.3. TMP and PF Inhibit Ox-LDL-Induced Tube Formation

**Figure 5:**
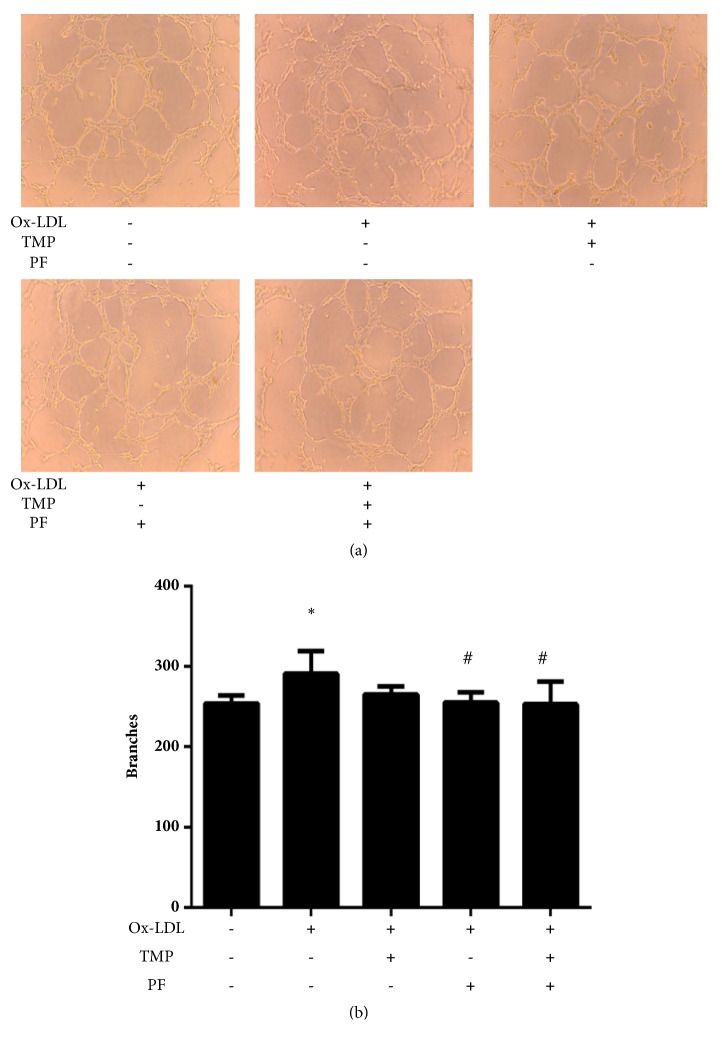
Effects of TMP and PF alone or in combination on ox-LDL-induced tube formation. (a) The network-like structures were examined under an inverted microscope (20×). (b) Quantitative analysis of the number of branch points in ox-LDL-induced HUVECs. Values are means ± SD. *∗P *< 0.05 versus control group; #*P *< 0.05 versus ox-LDL-induced group.

Quantitative measurements showed that ox-LDL significantly increased the numbers of tube branch points (*P*<0.05), while PF alone or in combination with TMP significantly decreased the number of tube branch points (*P*<0.05) (Figure 5). These results indicated that PF alone or in combination with TMP exhibited inhibitory effects on ox-LDL-induced tube-forming ability of HUVECs.

### 3.4. Effect of TMP and PF on the Ox-LDL-Induced Expression of CD31 and vWF

**Figure 6:**
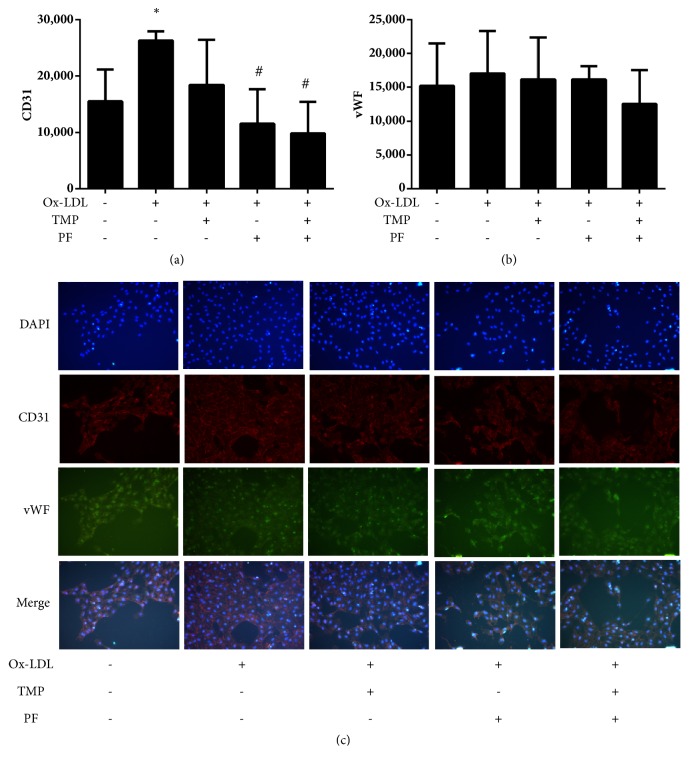
Effects of TMP and PF alone or in combination on CD31 and vWF expression in ox-LDL-induced HUVECs. Quantitative analysis of the IOD of (a) CD31 and (b) vWF. Values are means ± SD. *∗P *< 0.05 versus control group; #*P *< 0.05 versus ox-LDL-induced group. (c) The fluorescence intensity was examined under an inverted microscope (20×).

We observed a higher level of CD31 fluorescence intensity in ox-LDL-induced cells than in control cells (*P*<0.05), which indicated that ox-LDL promoted neovascularization. PF alone or in combination with TMP was found to significantly decrease the CD31 fluorescence intensity (*P*<0.05) and thus inhibit ox-LDL-induced angiogenesis. However, TMP and PF alone or in combination failed to produce a detectable effect on the expression of vWF (*P*>0.05) (Figure 6), which is a marker of mature vessels.

### 3.5. Effect of TMP and PF on the Ox-LDL-Induced Expression of Angiogenesis-Associated Protein

**Figure 7:**
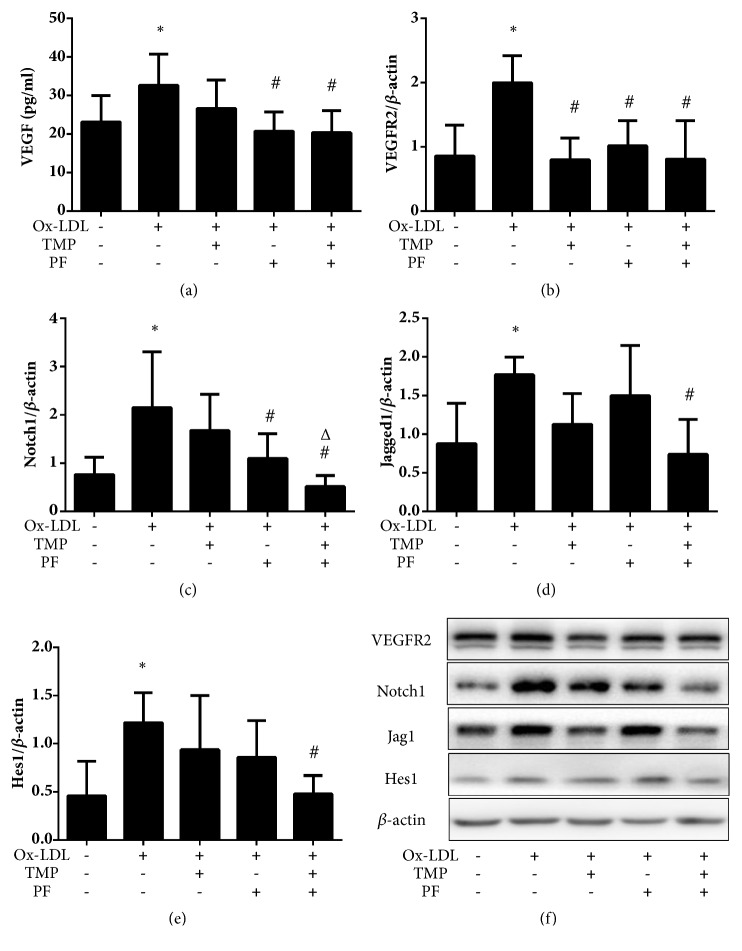
Analysis of angiogenesis-associated protein expression. (a) Secreted VEGF protein level in the supernatant of ox-LDL-induced HUVECs was analyzed by an ELISA. Protein expression levels of (b) VEGFR2, (c) Notch1, (d) Jagged1, and (e) Hes1 were analyzed by western blot. Values are means ± SD. *∗P *< 0.05 versus control group, #*P *< 0.05 versus ox-LDL-induced group, and Δ*P *< 0.05 versus TMP group. (f) Protein extracts were immunoblotted to determine the relative expression levels of VEGFR2, Notch1, Jagged1, and Hes1.

In our study, ox-LDL significantly increased the expression of VEGF, VEGFR2, Notch1, Jagged1, and Hes1 (*P*<0.05), thus indicating that ox-LDL-induced angiogenesis by activating the VEGF/VEGFR2 and Jagged1/Notch1 pathways. TMP downregulated VEGFR2 expression but did not regulate the expression of VEGF, Notch1, Jagged1 or Hes1; PF downregulated the expression of VEGF, VEGFR2, and Notch1 but did not regulate the expression of Jagged1 and Hes1. However, combination treatment not only decreased the expression of VEGF and VEGFR2 but also decreased the expression of Notch1, Jagged1, and Hes1 (*P*<0.05), thus indicating that an antiangiogenic effect of the combination of TMP and PF is mediated through the VEGF/VEGFR2 and Jagged1/Notch1 pathways. In addition, combination treatment with TMP and PF induced stronger inhibitory effects on the expression of Notch1 than did treatment with TMP alone (*P*<0.05) (Figure 7).

### 3.6. Inhibition by Combination Treatment and Pathway Inhibitor

**Figure 8:**
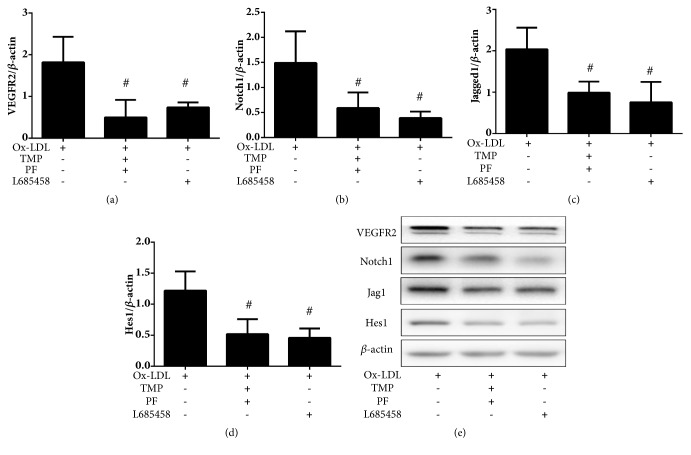
Analysis of the effects of combination treatment and Notch inhibitor treatment on ox-LDL-induced angiogenesis. Protein expression levels of (a) VEGFR2, (b) Notch1, (c) Jagged1, and (d) Hes1 were analyzed by western blot. Values are means ± SD. #*P *< 0.05 versus ox-LDL-induced group. (e) Protein extracts were immunoblotted to determine the relative expression levels of VEGFR2, Notch1, Jagged1, and Hes1.

To further examine the mechanisms by which changes in Notch1 and VEGFR2 expression alter angiogenesis upon combination treatment with TMP and PF, an inhibitor of the Notch signaling pathway was used. We found that Notch inhibitor not only inhibited the expression of Notch1, Jagged1 and Hes1, but also decreased the expression of VEGFR2 (*P*<0.05). Additionally, the ox-LDL-induced expression of VEGFR2 was found to be reduced more by combination treatment than Notch inhibitor. Thus, we demonstrated that combination treatment with TMP and PF potentiated an antiangiogenic effect through the suppression of the Jagged1/Notch1 pathway and the further decrease in the expression of both Hes1 and VEGFR2, and not just through the suppression of the VEGF/VEGFR2 pathway (Figure 8).

## 4. Discussion

**Figure 9:**
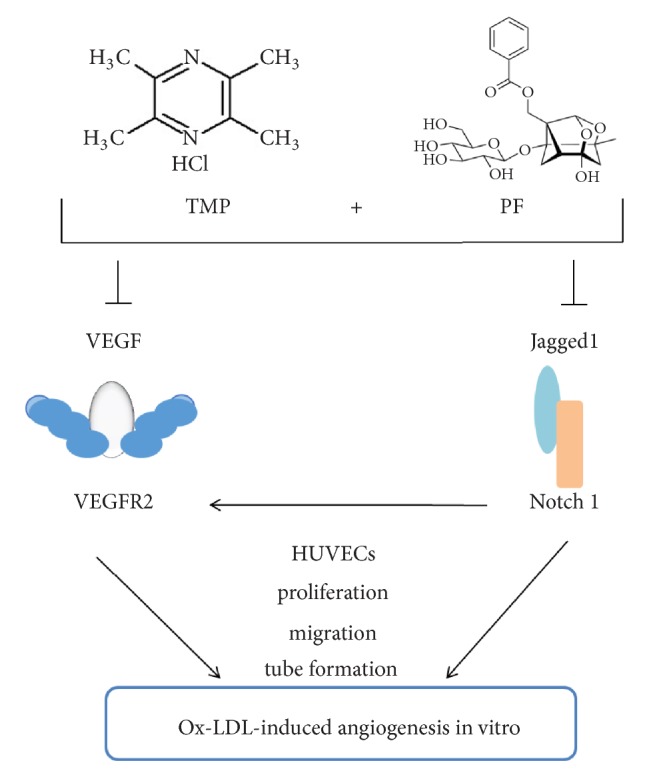
A schematic diagram of the proposed antiangiogenic mechanisms of combination treatment with TMP and PF in ox-LDL-induced HUVECs.

Angiogenesis contributes to plaque instability in atherosclerosis, and the inhibition of ox-LDL-induced angiogenesis might stabilize plaques [4]. Our findings revealed that the combination of TMP and PF exhibited an antiangiogenic effect on ox-LDL-induced HUVECs, and the inhibitory effect was associated with its suppression of the VEGF/VEGFR2 and the Jagged1/Notch1 signaling pathways. These results indicated that combination treatment with TMP and PF inhibited ox-LDL-induced angiogenesis with advantages of multiple targets and multiple pathways, which might stabilize plaques (Figure 9).

Previous studies have shown that combination treatment with TMP and PF might be a promising therapeutic strategy for ischemic disease and optimal compatibility ratio is TMP 1 *μ*mol/L and PF 10 *μ*mol/L [32]. We evaluated synergistic effect in cell proliferation and found the optimal compatibility ratio 1:10 for TMP and PF. These results suggest the potential of using lower TMP concentrations with higher PF concentrations to achieve an enhanced level of effectiveness. However, since the double-edged role of angiogenesis in myocardial ischemia and atherosclerosis [33], the effects of TMP and PF (1:10) on coronary atherosclerotic heart disease require further investigation. Moreover, the other compatibility ratios of TMP and PF could also be investigated in the future to observe different interactions.

Recent evidence has demonstrated that combination therapy could provide greater therapeutic benefits to atherosclerosis, which possesses complex pathophysiology and therefore is difficult to treat using single target approach [18]. Our study indicated that TMP inhibited cell proliferation and migration by decreasing the expression of VEGFR2; PF inhibited angiogenesis by mainly decreasing the expression of Notch1 and then decreasing the expression of VEGFR2; however, the combination of TMP and PF attenuated angiogenesis by inhibiting both the VEGF/VEGFR2 and Jagged1/Notch1 pathways. Therefore, combination treatment with TMP and PF exhibited an antiangiogenic effect through multiple targets and multiple pathways, which showed the advantages of combination therapy.

Notch1 selectively binds to Jagged1 under inflammatory conditions, and the Jagged1/Notch1 signaling pathway is then activated to promote angiogenesis in atherosclerosis [34, 35]. In the present study, TMP had no effect on the Notch pathway and did not significantly inhibit tube formation; PF decreased the expression of Notch1 and inhibited angiogenesis; combination treatment with TMP and PF significantly decreased the expression of Notch1 when compared with TMP treatment. These results indicated that Notch1 may be one of the major regulatory factors in the antiangiogenic effect of combination treatment. Nevertheless, an in vivo study using ApoE-/- mice would be preferred in the future to examine the role of the Notch pathway in the antiangiogenic effect of combination treatment.

Herb pairs as the basic composition units of Chinese herbal formulae are of special clinical significance in traditional Chinese medicine, and their feature of simplicity can facilitate studies on mechanism investigation [36]. Previous reports have shown that compound of active constituents of Ligusticum chuanxiong Hort. and Radix Paeoniae Rubra has inhibitory effect on plaque angiogenesis in vivo [13, 14]. Our study further examined the effect of TMP and PF on ox-LDL-induced angiogenesis in vitro and partly elucidated the antiangiogenic mechanism of Ligusticum chuanxiong Hort. and Radix Paeoniae Rubra herb pair. Nowadays, endothelial to mesenchymal transition (EndMT) makes a major contribution to the vascular remodelling and neointimal formation, which correlates with the unstable plaques and promotes atherosclerosis progression [37]. Hence, the effects of TMP and PF on EndMT and the connection between angiogenesis and EndMT require further investigation. Moreover, it should be noted that further clinical research is required for verifying the efficiency of Ligusticum chuanxiong Hort. and Radix Paeoniae Rubra herb pair, and more research is needed for the standardization and safety evaluation.

## 5. Conclusion

Our results demonstrated that combination treatment with TMP and PF had an antiangiogenic effect on ox-LDL-induced HUVECs by inhibiting the VEGF/VEGFR2 and Jagged1/Notch1 signaling pathways and thus might stabilize plaques and decrease the incidence of cardiac and cerebrovascular events. Therefore, these results may provide a new direction for future drug treatment strategies in atherosclerosis.

## Data Availability

The data used to support the findings of this study are available from the first author upon reasonable request (Email:yuanrong427@163.com).
